# Who Began It?

**Published:** 1913-05

**Authors:** 


### Who Began It?

Mamie had been naughty, and her mother finally had recourse to the time-honored remedy in such cases.

"Mamma," she sobbed, "did gran'ma spank you when you was little?"

"Yes, dear," said her mother; "she did when I was naughty."

"And did her mother spank her?"

"Yes."

"An' was she spanked, too, when she was bad?"

"Yes."

"Well, who started this blamed thing anyhow?"—*Ladies' Home Journal.*

